# Silver-Based SERS Pico-Molar Adenine Sensor

**DOI:** 10.3390/bios10090122

**Published:** 2020-09-11

**Authors:** Yonhua Tzeng, Bo-Yi Lin

**Affiliations:** Department of Electrical Engineering, National Cheng Kung University, Tainan 701401, Taiwan; 03132026@gm.scu.edu.tw

**Keywords:** SERS, silver, adenine, plasmon, Raman scattering, copper, pH

## Abstract

Adenine is an important molecule for biomedical and agricultural research and applications. The detection of low concentration adenine molecules is thus desirable. Surface-enhanced Raman scattering (SERS) is a promising label-free detection and fingerprinting technique for molecules of significance. A novel SERS sensor made of clusters of silver nanostructures deposited on copper bumps in valleys of an etched silicon substrate was previously reported to exhibit a low and reproducible detection limit for a 10^−11^ M neutral adenine aqueous solution. Reflection of laser illumination from the silicon surface surrounding a valley provides additional directions of laser excitation to adenine molecules adsorbing on a silver surface for the generation of enhanced SERS signal strength leading to a low detection limit. This paper further reports a concentration dependent shift of the ring-breathing mode SERS adenine peak towards 760 cm^−1^ with decreasing concentration and its pH-dependent SERS signal strength. For applications, where the pH value can vary, reproducible detection of 10^−12^ M adenine in a pH 9 aqueous solution is feasible, making the novel SERS structure a desirable pico-molar adenine sensor.

## 1. Introduction

Adenine is one of aromatic bases of DNA and RNA. Active interactions of adenine with silver- and gold-based sensors result in favorable surface enhanced Raman scattering (SERS) signal strength. SERS can detect adenine in a low-concentration aqueous solution, which regular Raman scattering technique cannot. To achieve this, adenine molecules must adsorb on a SERS sensor with very high plasmonic coupling induced local electromagnetic fields. An exciting laser beam causes electrons in a metal nanostructure to drift in resonance to the incident electromagnetic fields. High-concentration negative and positive charges accumulate on two closely spaced counter metal surfaces. These charges induce much stronger electromagnetic fields than that of the incident laser beam. Molecules which adsorb on counter surfaces in a nanoscale gap are therefore subjected to much stronger excitation than the laser beam alone and generate strong SERS signal strength for detection by a spectrometer [1,2]. High sensitivity adenine sensors are desirable for the detection of diseases, DNA hybridization, and biomedical and agricultural applications [3,4,5,6]. For the application of single-molecular SERS detection, “hot spots” are referred to as nano-gaps with less than 10 nm gap spacing [7]. When the gap spacing is smaller than the electron tunneling threshold, the effectiveness of plasmonic coupling decreases. When the gap spacing is too large, the plasmonic coupling induced local electromagnetic fields are less stronger than that of the optimal gap spacing. When silver nanocrystals grow larger laterally, the gap spacing between neighboring crystals decreases. For the SERS sensors, multiple silver nano-gaps jointly contribute to the SERS signal strength. The silver gap spacing ranges from zero to more than 10 nm. Therefore, in the following discussion, silver nano-gaps, where strong local electromagnetic fields are induced by laser induced plasmonic coupling to contribute significantly to the measured SERS signal strength, will be referred to as SERS “hot spots”. With irregular shaped and non-periodic distributed silver nanocrystals, which grow both laterally and longitudinally, it is reasonable to expect that some silver nanogaps are closer to the optimal gap spacing and “hotter” than the other, and a specific local SERS enhancement factor is larger than the remaining areas.

Interactions of SERS sensor with molecules affect where and how molecules adsorb on the sensor surface. Electrostatic coupling and functional groups assisted interactions are more active among complex coupling mechanisms. For example, adenine has an amino group, which shows positive potential and tends to interact with the silver surface by a nitrogen atom. The higher the probability for molecules to adsorb on SERS “hot spots” is, the lower a sensor might achieve its low detection limit. On the other hand, adsorption of molecules on the sensor surface where local electromagnetic fields are low is not desirable. Due to the different and complex interactions of different molecules with the sensor surface, the low detection limits for different molecules using the same SERS sensor may differ by orders of magnitude [8,9,10].

Adenine adsorbs on silver surface in different orientations depending on a number of complex parameters. In most reported cases, adenine molecules adsorb on silver metal at a tilted angle to the surface [11]. Some planar adsorption was also reported [12]. The signal strength of a specific SERS vibration mode and frequency depends on the alignment of the molecular structure with the exciting electromagnetic fields. We reported a novel means of graphene islands masked selective chemical plating technique for the depositing silver nanocrystals on copper [8,9]. Discrete but closely spaced silver nanocrystals were deposited on planar and curved copper surfaces to serve as high-sensitivity SERS sensors. Due to the different and complex interactions and adsorption mechanisms, the SERS detection limit for adenine molecules was found to be inferior to that for Rhodamine 6G molecules by many orders of magnitude using the same SERS sensor [12,13,14,15,16,17,18].

Even after we invented a novel technique to fabricate closely spaced silver nanocrystals on discrete copper bumps in the valleys of etched hole in a silicon substrate, the achievable lowest detection limit for adenine improved by several orders of magnitude to 10^−11^ M [9]. This is still several orders of magnitude worse than the low detection limit of 10^−16^ M we achieved for R6G molecules. Aiming at further improving the low detection limit of adenine, the ring-breathing mode vibration of adenine, which generates the strongest distinguishable SERS signal strength from the background signals are investigated and the pH value of the adenine solution in water is varied to influence the interactions of adenine molecules with the silver SERS sensor. As the pH value changes, the concentration of H^+^ ions carrying positive charges and that of OH^−^ ions carrying negative charges also vary accordingly. These ions react with adenine molecules and alter its coupling mechanism and strength with silver. The goal is to discover and demonstrate the best conditions for silver SERS sensor to detect adenine molecules at as low a concentration as possible.

In prior reports, the detection limit of adenine molecules by gold- and silver-based SERS sensors has been reported to range mainly from 10^−8^ M to 10^−9^ M [19,20,21,22,23]. Few research groups reported the detection of adenine molecules at a lower concentration of 10^−10^ M [24] and 10^−11^ M [25]. The low detection limit is usually achieved by 3D nano-scale structures or colloidal noble metal particles. We reported a 2D graphene masked silver SERS sensor, which is capable of detecting 10^−11^ M adenine [10].

## 2. Materials and Methods

The fabrication process for the silver SERS sensors for this research has been described in detail elsewhere [8,9,10]. In brief, holes are chemically etched into a planar silicon wafer [26]. Copper films are deposited by RF magnetron sputtering. During the copper annealing and rapid thermal CVD graphene process, the sputtered thin film copper melts, flows, and then solidifies at the valleys of etched silicon holes. Rapid thermal CVD of graphene on the copper bumps and residual copper on the silicon substrate creates a graphene template. Due to the low melting point and rapid vaporization of a copper thin film of 100 nm thick on silicon, a low temperature rapid thermal CVD of graphene is carried out at 900 °C [10], which is lower than the typical thermal CVD on thick copper foil at 1000 °C [8]. Discrete graphene nano-islands of high density and high crystallinity are formed on copper foils at 1000 °C to serve as a template [10]. At the CVD temperature of 900 °C, the graphene nucleation density and the defect density of graphene domains are high [8]. The selective growth of silver nanocrystals occurs by chemical plating on exposed copper surfaces and copper surface covered by defective graphene. On copper bumps, silver crystals grow perpendicular to the curved surface of copper bumps to form clusters of elongated nanocrystals pointing in different directions. In the chemical plating of silver on copper, copper metal dissolves in the silver nitrate solution and silver deposits on the copper surface [27]. Silver does not deposit on graphene surface.

Adenine (Sigma, purity > 99%) of 0.5 mg is dissolved in deionized water to make 3.7 × 10^−6^ M adenine in water solution. We will refer to this as the 10^−6^ M solution in the following discussion. We dilute this adenine solution further by 10 times repetitively by adding deionized water to prepare a series of adenine water solution of different concentrations. We remove the sensor from the solution and blow it dry by air after immersing it in adenine water solution for 10 min.

A Horiba Scientific Raman system with a green laser at 532 nm and laser power at 450 mW is used to measure Raman spectra. The laser beam is focused on the sensor surface in an area of about 10 µm in size. SERS spectra are measured in several different areas on each sensor for each concentration of adenine water solution. The concentration of adenine is considered to be above the low detection limit if the ring-breathing mode SERS peak around 760 cm^−1^, which is characteristic of adenine molecules [28], this is clearly distinguishable from the background signal. The low detection limit is determined by the lowest concentration of adenine solution, which produces a clearly detectable adenine SERS scattering peak. Multiple sensors are characterized to confirm the reproducibility.

For adjusting the pH value of adenine water solution below 7, HCL is added. For a pH value higher than 7, NaOH is added to the adenine water solution. The test paper for the pH value is purchased from Adventec for exhibiting different colors based on different pH values.

## 3. Results and Discussion

### 3.1. Silver Crystals on Copper Bumps under Laser Illumination in Multi-Directions

**Figure 1:**
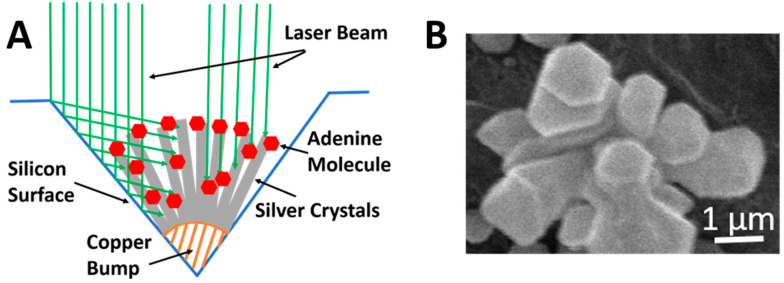
(**A**) Schematic diagram of multi-directional laser illumination of silver crystals on a copper bump. (**B**) SEM image of silver crystals grown on a copper bump.

Figure 1A is a schematic diagram of multi-directional laser illumination of silver crystals on a copper bump inside a silicon hole. Figure 1B shows an SEM image of elongated silver crystals grown on a curved copper bump forming nanoscale gaps between crystals suitable for laser induced plasmonic coupling effects for SERS applications. As shown in Figure 1A, a laser beam aiming at the cluster of silver crystals results in multi-directional illumination of adenine molecules adsorbing on silver. The orientation in which an adenine molecule adsorbs on silver varies depending on the interactive mechanism between adenine and silver. Polarization and orientation of laser illumination thus cause the SERS spectra of adenine to vary, too. With silicon surface surrounding the silver cluster serving as mirrors, silver crystals and adenine molecules on silver are subjected to multi-directional laser illumination. The most favorable combination of laser intensity and orientation of illumination generates the strongest SERS signal strength from a small amount of adenine molecules in areas with strong local electromagnetic fields. In the case of this SERS sensor, the strongest SERS signal strength originating from the ring breathing mode vibration of low-concentration adenine in aqueous solution is at 760 cm^−1^ when the SERS is under illumination by a green laser beam at 532 nm. This ring-breathing mode SERS peak of adenine shifts significantly from the original Raman peak of adenine at 721–726 cm^−1^ and SERS peak around 745 to 760 cm^−1^ depending on the adenine concentration and other conditions of measurements.

The effectiveness of excitation of the ring-breathing mode vibrational Raman scattering is dependent on the polarization of plasmonic coupling induced strong local electromagnetic fields with respect to the orientation of adenine adsorption on silver. The strength of local electromagnetic fields depends on the polarization of the incident laser beam with respect to the short gap distance between two silver nanocrystals. When the electromagnetic (electric) field of the illuminating laser beam is in aiming between two silver nanocrystals along the short nanogap, the plasmonic effect is the most effective and the plasmonic coupling induced local electromagnetic field is the highest. Therefore, the strong SERS signal strength is detected from molecules, which adsorb on silver inside the nanogap.

Adenine molecules adsorb on silver in a variety of orientations with respect to the silver surface. The silver nanocrystals also grow in different directions with respect to the incident laser beam from the curved copper bumps. Therefore, each adenine molecule experiences different optimal orientations of light illumination in order to generate the strongest possible SERS signal. When the concentration of adenine is very low and there is only one direction of light illumination, the chance for the light illumination to match perfectly the local silver structure for effective plasmonic coupling is low. If it does not match the needs for optimal plasmonic coupling for the silver nanocrystal, the adenine molecule on the silver nanocrystal may not produce a high enough SERS signal strength for the detectability of the molecule. Therefore, multi-directional light illumination, including reflected light from the side silicon walls, increases the probability of few and individual molecules from a very low concentration adenine aqueous solution to have optimal or near optimal light illumination. Collectively, few adenine molecules from a very low concentration adenine aqueous solution suffice to generate a stronger SERS signal strength than the background signal and the adenine is detectable by the SERS sensor.

In reference [29], the importance of growing silver crystals, which are close to each other but do not overlap with each other was pointed out. A chemically controlled growth process for silver hexagonal columns was applied to achieve it. In this paper, closely spaced but discrete graphene islands or graphene films with high density of defects are applied as masks for selective growth of silver crystals with separated roots on copper so that they do not overlap with each other.

### 3.2. Adenine Molecular Structures and Raman and SERS Spectra

**Figure 2:**
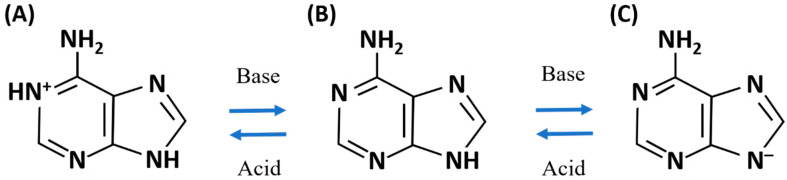
Examples of molecular structures of (**B**) neutral adenine, (**A**) positively charged adenine in alkaline, and (**C**) negatively charged adenine in acid.

**Figure 3:**
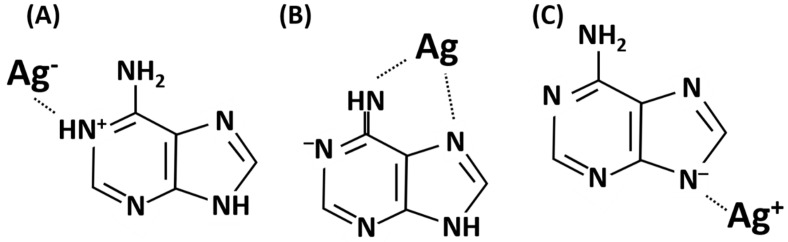
Schematic diagrams of adenine adsorption on silver. (**A**,**C**) Electrostatic interactions of adenine with silver and (**B**) interactions by functional groups of adenine molecules with silver.

Figure 2 shows the molecular structures of (B) neutral adenine, (A) positively charged adenine in acidic water solution, and (C) negatively charged adenine in alkaline water solution. Adenine interacts with the silver SERS sensor by both electrostatic forces and interactions of functional groups as shown in Figure 3A–C, respectively [30]. Strong interactions between adenine and silver on SERS sensor surface and the adsorption of adenine at silver “hot spots” contribute to the measured SERS scattering signal strength. Therefore, by properly controlling the charge states of adenine, the previously achieved low detection limit of 10^−11^ M adenine aqueous solution is expected to improve further.

**Figure 4:**
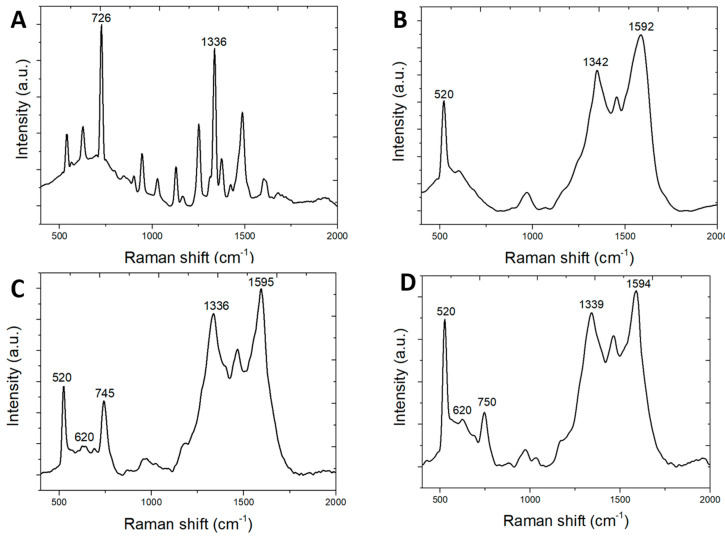
Raman and surface-enhanced Raman scattering (SERS) spectra. (**A**) Raman spectrum of adenine powder used for this study. (**B**) SERS spectrum of deionized water without adenine. (**C**) SERS spectrum of adenine powder wetted by deionized water. (**D**) SERS spectrum of saturated concentration of adenine in water.

Figure 4A shows a Raman spectrum of dry adenine powder on a flat silicon dioxide substrate. The ring-breathing mode vibration Raman peak is the strongest one at 726 cm^−1^. Figure 4B shows the SERS spectrum of the SERS sensor without adenine. The SERS sensor is first immersed in DI water without adenine. It is then blown dry in air before the SERS spectrum is measured. This spectrum serves as a reference to distinguish adenine peaks from those from the sensor itself. The main peaks are the silicon peak at 520 cm^−1^, the D-band of graphene at 1342 cm^−1^, and the G-band of graphene at 1592 cm^−1^. The silicon peak at 520 cm^−1^ is used as a calibration reference for the SERS peak position. There are no strong SERS peaks in the range of 700–800 cm^−1^. The adenine ring-breathing peak will appear in this region if the water solution contains adequate adenine. Figure 4C shows a SERS spectrum of adenine powder wetting by DI water on the SERS sensor. The ring-breathing peak shifts from 726 to 745 cm^−1^ due to the adenine adhesion orientation effects on silver and plasmonic coupling effects of the SERS sensor. Figure 4D shows a SERS spectrum of saturated adenine water solution. Adenine is added to DI water for the maximum adenine to dissolve in water at room temperature. Excessive adenine will remain at the bottom of the water solution. A sample is taken from the water solution for the SERS sensor to measure the SERS spectrum. For the adenine water solution of a high concentration, the adenine ring-breathing SERS peak shifts further to 750 cm^−1^. The adenine ring-breathing SERS peak shifts further to around 760 cm^−1^ when the adenine water solution is diluted by DI water. This 760 cm^−1^ peak will be used to determine the detection of adenine.

Reference [31] reported that the Raman wavenumber shift at 720 cm^−1^ is due to reduced bond distance between the silver atom and the N3 nitrogen of adenine. The extent of SERS shift observed in this work is much larger than what was reported in the reference.

### 3.3. Effects of pH Value on SERS Spectra of Adenine

**Figure 5:**
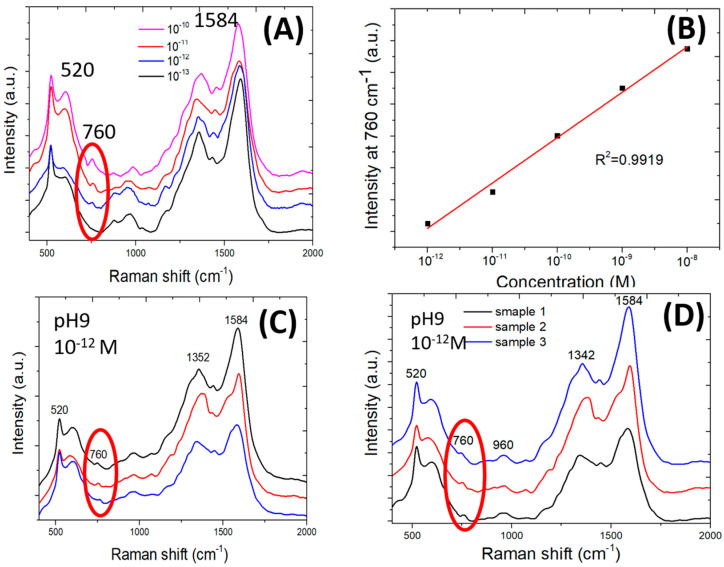
Raman spectra and intensity of Raman scattering signal. (**A**) Raman spectra of adenine of varied concentration in pH 9 water solution. (**B**) Raman scattering signal strength of adenine as a function of the adenine concentration. (**C**) Three Raman spectra measured at three different areas on a SERS sensor for 10^−12^ M adenine in pH 9 water solution. (**D**) Three Raman spectra measured using three different SERS sensors for 10^−12^ M adenine in pH 9 water solution.

Figure 5A shows the Raman spectra of adenine of 10^−10^–10^−13^ M concentration in a pH 9 water solution. The adenine ring-breathing peak shifts with its adsorption orientation on silver and its interactions with silver surface [32,33]. The ring-breathing mode SERS peak for low-concentration adenine in water is measured to be around 760 cm^−1^. The SERS peak at 760 cm^−1^ which is circled in red indicates that adenine in a water solution with pH 9 has been detected at as low concentration as 10^−12^ M. The 760 cm^−1^ peak is not detectable from the SERS spectrum measured from 10^−13^ M adenine. The SERS signal strength at 760 cm^−1^ is plotted as a function of the adenine concentration and shown in Figure 5B. Figure 5C shows that the 760 cm^−1^ SERS peak is detected by all SERS spectra measured at three different surface areas on the same sensor using a pH 9 water solution of 10^−12^ M adenine. Figure 5D shows that the same low detection limit is reproducible by three different sensors.

**Figure 6:**
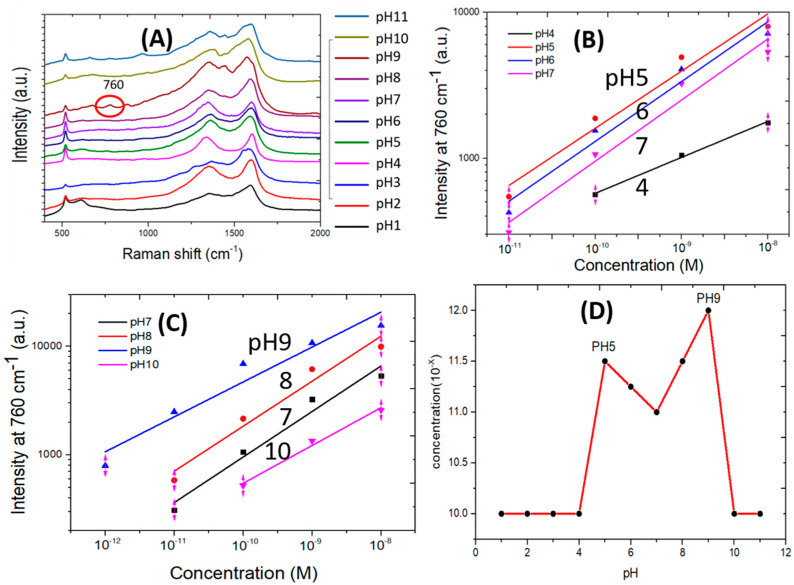
(**A**) SERS spectra measured for 10^−12^ M adenine water solution of different pH values. (**B**,**C**) Adenine SERS signal intensity as a function of adenine concentration and pH value. (**D**) Low detection limit of adenine concentration by the SERS sensor in study in water solution of different pH values.

Figure 6A shows SERS spectra measured from the 10^−12^ M adenine water solution at pH values ranging from 1 to 11. The adenine SERS peak at 760 cm^−1^ is detectable only at pH 9. The SERS scattering signal strength at 760 cm^−1^ measured from the adenine solution in water versus adenine concentration is shown in Figure 6B,C for different pH values. Log-log charts are applied for easy distinguishing of the small signal strength measured at different pH values from low concentration adenine. Data sets in Figure 6B,C show a nonlinear log-log relationship. Least squares regression is applied to find linear approximation for each set of data. These lines help show the magnitude and trend of SERS signal strength at low concentration and thus the low detection limits. The highest SERS scattering signal strength is measured from the adenine solution in water of pH 9. As shown in Figure 4 and Figure 5, at pH 9, some adenine molecules become negatively charged due to reactions of neutral adenine with abundant OH^−^ ions in the solution. Interactions by functional groups with silver and electrostatic coupling between negatively charged adenine and positively charged silver surface jointly enhance the adsorption of adenine on closely spaced silver nanoparticles. The sensor surface surrounding the silver nanocrystals and on the remaining sensor surface is covered by graphene. Adenine is more likely to adsorb on the silver surface by strong interactions than on the graphene covered silicon substrate.

Figure 6B shows the SERS signal strength of adenine in water of the same concentration. The SERS signal intensity increases from pH 7 to 6, reaches the maximum at pH 5 and then decreases with further decrease in pH value. Additional electrostatic coupling by positively charged adenine promotes the adsorption of adenine.

Figure 6C shows the SERS scattering signal strength of adenine of the same concentration in water. The SERS signal intensity increases from pH 7 to 8, reaches the maximum at pH 9 and then decreases with further increase in the pH value. Additional electrostatic coupling between negatively charged adenine and silver is believed to promote the adsorption of adenine on silver “hot spots” for the generation of high measured SERS scattering signal strength. The extent of increase in SERS signal intensity of adenine from pH 7 to 9 is more than that from pH 7 to 5.

Figure 6D summarizes the low detection limit of the SERS sensor as a function of the pH value of adenine water solution. The lowest detection limit for adenine occurs at pH 9 to be 10^−12^ M. For pH values between 5 and 8, the low detection limit is one order of magnitude higher than that at pH 9. For pH values lower than 5 and higher than 9, the low detection limit is two orders of magnitude higher than that at pH 9. In strong acid and base, both the adenine molecules and the SERS sensor substrate are believed to have been altered to some extent to cause the SERS signal strength to decrease significantly from that measured between pH 5 and 9. A pico-molar sensor for adenine water solution at pH 9 has been demonstrated using a SERS sensor with clusters of silver nanoparticles grown on discrete copper bumps where copper is not fully covered by a graphene mask and is partially exposed to the chemical plating solution.

Adsorption fashion of adenine on silver surface is a complex problem. Computational results are often compared with and validated by experimental data to reveal the most likely fashion of molecular adsorption. We hope that experts in computational biosensors will become interested in this problem after reading this paper.

The charge state of clusters of silver on micron sized copper bumps inside etched holes is more difficult to characterize than that of a planar silver surface. It is an important information to know. We hope that readers of this paper who have suitable expertise and experimental facilities can publish their findings in this regards. It shall help further improvement of the low detection limit and reproducibility of this SERS sensor in the future.

### 3.4. Effects of Adenine Concentration and Reproducibility of SERS Spectra

**Figure 7:**
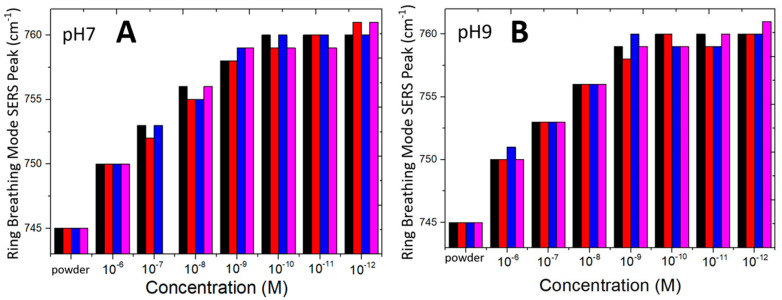
Bar charts of four SERS sensors (displayed in different colors) exhibiting a similar trend of shift of the ring-breathing mode SERS peaks of adenine. Measured SERS peaks of adenine powder on the sensor are also included for comparison. (**A**) pH 7 and (**B**) pH 9 aqueous solution.

The ring-breathing mode of adenine vibration at low concentration and under conditions of this SERS sensor shifts so significantly from the commonly measured Raman spectra and SERS spectra using different SERS structures that it is worthy of a more detailed measurement of the continuous shifting from medium to very low concentration. Raman spectra of adenine powder on a flat silicon substrate is first confirmed to exhibit a commonly reported ring-breathing mode of adenine at near 726 cm^−1^ under excitation by a green laser at 532 nm. When a small amount of adenine powder is placed on the surface of a SERS sensor under study in this work, the SERS peak shifts from the regular Raman peak at 726 to 745 cm^−1^. SERS spectra measured from the 10^−6^ M adenine aqueous solution displays the ring-breathing mode vibration at around 750 cm^−1^. Figure 7A shows that the SERS ring-breathing mode vibration measured from 10^−6^ M adenine is about 750 cm^−1^ and the peak shifts to 760 cm^−1^ when the concentration decreases to below 10^−9^ M adenine at pH 7. Four SERS sensors are used for the measurements with results being displayed in different colors. The pH value has little influence on the shift of the SERS peak. Figure 7B shows the shift of the SERS peak with concentration of adenine in pH 9 aqueous solution.

**Figure 8:**
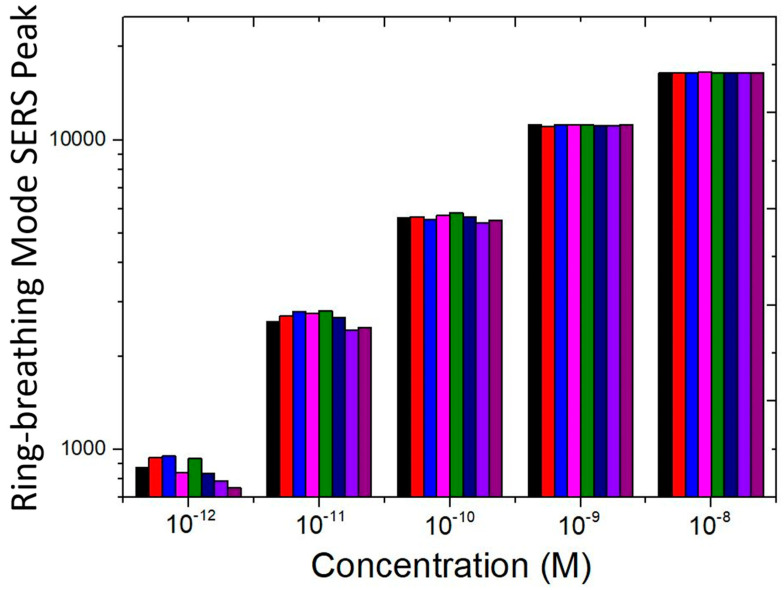
Reproducibility of SERS signal strengths of the ring-breathing mode vibration measured by eight SERS sensors (displayed in different colors) from pH 9 adenine aqueous solution.

Figure 8 displays the measured signal strength of the ring-breathing mode SERS peak from pH 9 adenine aqueous solution. Eight SERS sensors are used to measure the signal strength. Measured results from each sensor is shown in different colors. It should be noted that although the combined bar chart looks linear, if one focuses on just one color, for example, purple color in the bar chart of Figure 8, it would be clear that it does not represent a linear trend. The combined eight parallel bar charts in one graph visually misleads one to feel that it is linear. It is shown that the reproducibility is excellent for measuring 10^−9^ M and higher-concentration adenine aqueous solution. The fluctuation in measured SERS signal strength for lower-concentration adenine aqueous solution is due to the small amount of adenine molecules on the SERS sensor. Non-uniform distribution of a small amount of adenine molecules adsorbing at different parts of a sensor with varied strength of local electromagnetic fields and in varied orientations with respect to the silver surface is attributed to the increasing fluctuation. Nevertheless, all eight SERS sensors exhibit the low detection limit of 10^−12^ M adenine aqueous solution at pH 9. For applications, which allow the variation of the pH value, the low detection limit of the SERS sensor can be improved further beyond the very low detection limit of 10^−11^ M adenine in neutral aqueous solution by ten times and reaching 10^−12^ M at pH 9 and between 10^−11^ M and 10^−12^ M at pH 5.

The reproducible and concentration dependent adenine SERS shift has additional applications. For example, by measuring the SERS peak position of the adenine ring-breathing mode vibration signal using the same SERS sensor, it is possible to determine the concentration of the adenine in the concentration range where the SERS shift depends significantly on the concentration. On the other hand, it is a common limitation to SERS sensors that in order for a high-sensitivity SERS sensor to detect a specific molecule, there can be no interfering SERS peaks from other co-existing molecules. Otherwise, the interfering molecules must be filtered out by some means in advance.

Adenine’s ring-breathing mode vibration SERS peak is in a low-background range of the spectrum. The Raman bands of graphene does not affect the low detection limit of adenine adversely. For detecting other molecules, graphene can be etched first.

## 4. Conclusions

SERS sensors capable of detecting adenine in 10^−12^ M aqueous solution have been demonstrated. Chemically plated silver nanocrystals selectively grown on graphene masked copper bumps in valleys of chemically etched holes in a silicon substrate are used as SERS sensors. Adenine molecules adsorbing on closely packed silver crystals are subjected to laser excitation in multiple directions in the aid of light reflection from the silicon surface surrounding the valley. The combined effects of the high-density nanoscale gaps between silver crystals, the orientations of adenine adsorbing on silver, and the multi-directional laser excitation produce a strong ring breathing mode SERS signal, which shifts with decreasing adenine concentration towards 760 cm^−1^. The adsorption of adenine on silver is affected by the pH value of the aqueous solution. At pH 9, the SERS sensor is capable of reproducibly detecting 10^−12^ M adenine making it a desirable pico-molar adenine sensor. At pH 5, the low detection limit is between 10^−11^ M and 10^−12^ M.
